# Foreign Language Effect on Dishonesty

**DOI:** 10.3389/fpsyg.2021.633016

**Published:** 2021-10-28

**Authors:** Xiaolan Yang, Li Li, Rui Li

**Affiliations:** ^1^School of Business and Management, Shanghai International Studies University, Shanghai, China; ^2^Key Laboratory of Applied Brain and Cognitive Sciences, Shanghai International Studies University, Shanghai, China; ^3^School of Economics and Finance, Shanghai International Studies University, Shanghai, China

**Keywords:** foreign language, dishonesty, cognitive load, die-rolling task, sender-receiver game

## Abstract

The purpose of this paper is to investigate whether the use of foreign languages affects individuals’ dishonesty. We recruited native Chinese speakers who can speak English as a foreign language at universities in China, and they were randomly assigned to a native language (NL) or foreign language (FL) condition. Participants in each condition were required to finish the same tasks, in which they would benefit more from lying; the tasks were administered in either Chinese or English. We conducted one die-roll game in Study 1 and one cheap-talk sender-receiver game in Study 2. In both Study 1 and Study 2, we found that the proportion of lying was significantly lower in the FL condition than in the NL condition. Our results imply that the FL effect on dishonesty may be due to the cognitive load of communicating in a FL.

## Introduction

Dishonesty is a pervasive human behaviour that has greatly increased social costs in the economic and financial fields (Ederer and Fehr, 2007). Common examples of these increased costs include Internet fraud, corruption and tax evasion. Dishonesty usually manifests as the disposition to lie, cheat, fraud or deceive (Bucciol and Montinari, 2019). Lying is regarded as a form of dishonesty with the aim of instilling false belief in the victim and doing so intentionally by asserting what one believes is false (Adler, 1997). Understanding how people make decisions about whether to be dishonest has important implications for individuals and society.

With increases in international cooperation, communication between people who speak different languages is becoming more prevalent. Thus, decision making and moral judgement in foreign language environments are becoming the new normal. The first study to investigate the process differences between decision making in one’s native language (NL) and that in a foreign language (FL) was conducted by Keysar et al. (2012). They found that thinking in a FL reduces decision bias, and they named this phenomenon the FL effect. Inspired by this finding, many subsequent studies found that the FL effect is robust and is not restricted to risky decision making (Costa et al., 2014a; Gao et al., 2015; Hadjichristidis et al., 2015; Winskel et al., 2016) but also extends to decision making in other areas, including moral judgements (Cipolletti et al., 2015; Geipel et al., 2016; Hadjichristidis et al., 2017). People are more inclined to make utilitarian choices under conditions that require FL use (Costa et al., 2014b; Cipolletti et al., 2015; Corey et al., 2017). Researchers refer to this phenomenon as the moral FL effect.

Although research on the perceived trustworthiness of people speaking in their native or non-native language is fairly common (Cheng and Broadhurst, 2005; Lev-Ari and Keysar, 2010; Evans and Michael, 2014; Li, 2017), only a small number of studies has focused on the FL effect on lying and whether people lie less in a non-native language than in a native language (Suchotzki and Gamer, 2018; McDonald et al., 2020). Most of these studies compared deception behaviour in a NL condition versus FL condition, but with mixed findings. Some researchers observed that deception was more successfully and accurately detected in a NL than a FL (Da Silva and Leach, 2013; Leach and Da Silva, 2013; Elliott and Leach, 2016; Akehurst et al., 2018; Bereby-Meyer et al., 2020), whereas some researchers found the opposite to be true (Evans et al., 2013, 2017). Other research revealed no difference in the two language conditions (Cheng and Broadhurst, 2005; Caldwell-Harris and Aycicegi-Dinn, 2009; Evans and Michael, 2014; Duñabeitia and Costa, 2015; Alempaki et al., 2020).

Two research theories, cognitive load and emotional distance, can explain the differences between lying/truth telling in a NL and that in a second language. However, the two theories lead to competing conclusions.

Lying is cognitively more demanding than truth telling (Zuckerman et al., 1981; Vrij et al., 2008). The cognitive load associated with communicating in a FL is especially burdening for lying, and increasing cognitive load seems to make lying more difficult (Blandón-Gitlin et al., 2014), thereby further increasing the difference between lying and truth telling (Vrij and Granhag, 2012). Studies have found that people respond slower to lying in a FL than to lying in their NL, and the latter results in a stronger skin conductance response (Suchotzki et al., 2015; Suchotzki and Gamer, 2018), greater pupil dilation and a longer duration of pronunciation (Duñabeitia and Costa, 2015). Nonetheless, although most research has supported the cognitive load hypothesis, Blandón-Gitlin et al. (2017) argued that lying is not always more cognitively difficult than telling the truth.

The emotional distance hypothesis predicts that the reduced emotionality associated with FL use may facilitate lying. Emotional stimuli elicit less pronounced autonomic nervous system activity when presented in a FL (Harris et al., 2003). Moreover, advertisements are judged to be less emotional when presented in a FL (Puntoni et al., 2009). People are less aroused by emotionally laden expressions, such as childhood reprimands or taboo words in a FL than in their native tongue (Colbeck and Bowers, 2012). Diminished emotional arousal can facilitate lying and thereby reduce behavioural and autonomic differences between lying and truth telling.

Although they support different conclusions, the theories of cognitive load and emotional distance are not independent of each other. Some researchers found smaller RT differences between lying and truth telling in a FL compared to the NL of individuals (Suchotzki and Gamer, 2018; Frank et al., 2019). Their result could be explained by the antagonistic effect of cognitive load and emotional distance on lying, namely, the stronger emotional distance ‘cancelling out’ the increased cognitive load while lying in the FL (Suchotzki and Gamer, 2018).

Based on two experimental behavioural studies, this paper explores whether different languages modulate lying behaviours. In the first study, we used a die-rolling task similar to that of Fischbacher and Föllmi-Heusi (2013) and Bereby-Meyer et al. (2020) to investigate the FL effect on lying behaviour. In the task, each participant was asked to report the outcome of a six-sided die roll that only the participant who rolled the die could see. Their payoff depends on the reported roll of the die. The participants have an incentive to lie and report higher numbers to get a higher payoff. This methodology could measure the propensity of people to be dishonest. Then, in the second study, participants were required to take part in a cheap-talk sender-receiver game proposed by Gneezy (2005), where two participants were randomly paired into groups where the sender could benefit more from telling lies to the receiver.

Two different tasks were adopted separately in the two studies, as they can complement each other in comparing lying behaviour in different languages. In the die-rolling task, since each number outcome should happen with the same probability (1/6), we can compare the reported die-roll outcome at the aggregate level with the 1/6 benchmark to infer the propensity to lie at the group level, thus considering the NL and FL groups separately. Unlike the die-rolling task measuring aggregate-level dishonesty, the cheap-talk sender-receiver game enables us to utilise individual-level data to analyse lying behaviour (Gao et al., 2018). In addition, social interaction is involved in the cheap-talk sender-receiver game. Therefore, guilt, nervousness and other emotions related to lying might be more easily aroused by the cheap-talk sender-receiver game than by the die-roll task.

Based on the cognitive load hypothesis, using a FL will increase cognitive load and make lying more difficult. We may observe less cheating in the FL condition than in the NL condition in our two studies. However, according to the emotional distance hypothesis, using a FL will reduce emotionality associated with lying, e.g. guilt. We can predict that participants will cheat more in the FL condition than in the NL condition, especially in Study 2.

We will test those two hypotheses with opposite predictions about the FL effect on dishonesty. Our studies may offer a contribution by providing more reliable evidence of this effect. Previous studies on the FL effect on dishonesty have generally yielded competing conclusions, possibly because the results are based on different tasks. In a recent study, Bereby-Meyer et al. (2020) asked participants to roll a die and self-report the results to receive compensation. Using a FL (Hebrew, Korean or Spanish) to report the total die roll resulted in significantly lower earnings than using one’s native language (English), which suggests that speaking in a FL reduces lying behaviour. By contrast, Alempaki et al. (2020) designed a performance-difference-reporting game in which participants could inflate their relative performance in a real effort task, and they did not find a FL effect in lying behaviour. In their experiment, the FL was English, and the NL was Chinese or German. In the present study, we investigate whether we can obtain consistent results in two different typical tasks under the same language pairs, namely, Chinese and English.

## Study 1: Reporting the Outcome of a Die Roll

### Participants

A total of 106 undergraduate students (76 female) at Shanghai International Study University were recruited as participants *via* advertisements posted on the Internet. The average age of the participants was 20.06years old. All the participants were native Chinese speakers with more than 6years of experience speaking English as a FL. Among them, 43 students were language majors, and the remaining participants were economics, management, news communication, political science, law or other majors. The participants were compensated RMB 5 for participating and received an additional monetary reward based on their self-reported die-roll result. Questionnaires about the participants’ demographic information and language ability were conducted after the experiment. The participants were randomly assigned to the NL condition (*n*=49) or the FL condition (*n*=57).

On the questionnaires conducted after the experiment, the participants rated their English ability on a 4-point scale, with 4 indicating full fluency (*M*=2.70, *SD*=0.69). English proficiency did not differ significantly between the participants in the NL condition or FL condition (Mann-Whitney test, *p*=0.44). In addition, the participants in the FL condition were asked to evaluate their understanding of the experimental task on a 4-point scale, where 1 indicated ‘absolutely do not understand’ and 4 indicated ‘absolutely understand’.^1^ Statistical analysis of the data with a one-sample t-test revealed that the participants’ scores (*M*=3.56, *SD*=0.07) were significantly higher than 3 (*t*=7.93, *p*<0.001), indicating that they understood the decision task under FL conditions well.

### Materials and Procedure

#### Experimental Task

Study 1 was inspired by Fischbacher and Föllmi-Heusi (2013). In the experimental task, a one-shot individual decision-making task, the participants are required to roll a six-sided die in a cup and report the outcome of their first throw privately. The participants learned that the numbers they reported determined their payoff, as shown in Table 1 (100 points=RMB 1.5).

**Table 1:** Experimental payoff.

Number reported	1	2	3	4	5	6
Payoff (points)	30	60	90	120	150	0

In this task, the participants had an incentive to be dishonest and report higher numbers (except six) to obtain a higher payoff. However, the case differed for the number 6, which yielded the lowest payoff despite being the highest possible result. Participants who rolled a 6 could feel that the compensation was unfair and be tempted to correct for this unfairness by reporting a lower number.

Since the true outcomes of the die roll were revealed only to the participants themselves, lying could not be detected at the individual level; however, the true distribution of the outcome under full honesty is known. If all the participants are honesty, the outcomes will be consistent with the normal probability rule, namely, the proportion of each number will be 16.7% (1/6). If the participants tend to report a number higher than they actually rolled for a higher payoff, higher numbers appear with a higher probability. Hence, it is possible for us to evaluate lying behaviour of the participants in the FL group and the NL group separately.

#### Experimental Procedure

All participants were randomly assigned to one of the two conditions. Altogether, 57 participants (73.68% female) were assigned to the FL condition, and 49 (69.38% female) were assigned to the NL condition.^2^ A pen-and-paper experiment was conducted in classrooms at the university by a bilingual experimenter. The entire experiment, including the experimental instructions, interactions with the experimenter, and the questionnaires, was conducted in the assigned language. The participants rolled the die in an opaque cup and were the only ones who could see the results. Then, they were required to write down the result of the roll. After the experiment, we collected demographic data to confirm the eligibility of the participants.

### Result

Table 2 provides detailed information about the data collected in the die-rolling task. Columns 3–8 report the proportion of each number reported across the two conditions. In the task, as each number outcome should happen with the same probability (1/6), if all participants choose to tell the truth, the distribution of reported numbers in one group should be consistent with a uniform distribution. We compared the distribution of reported numbers in each condition to a uniform distribution and report the *p*-values in the second column. The results show that the distribution of numbers reported is significantly different from a uniform distribution only in the NL condition. Thus, we find evidence of lying in the NL condition but not in the FL condition. Using one’s NL thus increases the proportion of lying behaviour.

**Table 2:** Proportion of each number reported by condition.

Condition	p-values	Number reported (%)					
		1	2	3	4	5	6
Native language (NL)	<0.001	14.29	8.16	16.33	20.41	40.82	0
Foreign language (FL)	0.62	15.79	15.79	14.04	21.05	10.53	22.81

*The* p*-values reported in the second column are obtained by the chi-square goodness-of-fit test against a uniform distribution*.

**Figure 1:**
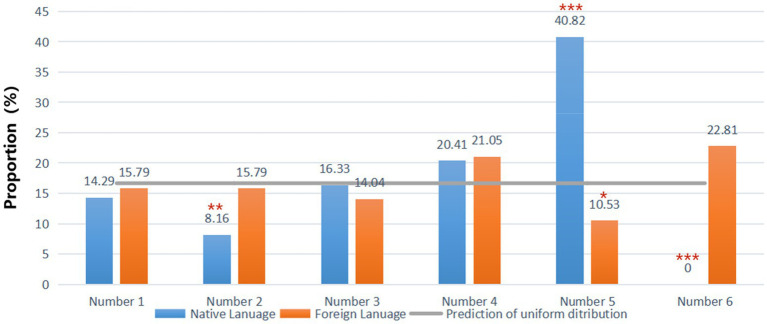
Percentage of reported number of participants by groups. Stars display the significance of two-sided binomial test that the observed percentage differs from 16.7%; ^*^10% level, ^**^5% level and ^***^1% level.

Figure 1 shows the proportion of each number reported by participants. According to Figure 1, the proportion of reported 5s was significantly higher than the prediction of a uniform distribution in the NL condition (two-sided binomial test, *p*<0.001). As there is no cost for lying for people who are homo economicus type, he/she would always report a 5 which yields the highest payoff. Besides, people who reported 6 which yields zero payoff are honest people. The fraction of honest people in the FL is much higher than in the NL. Another interesting observation is that the percentages of number 4 is not significantly above the expected 16.7% (two-sided binomial test, *p*=0.35) in the NL group. That is also true for number 4 reported in the FL group (two-sided binomial test, *p*=0.18). The results show that people might tend to report 5 rather than 4 if they decided to lie. In other word, most of the lying participants lie maximally in the NL.

To further verify the impact of using a FL on individual lying behaviour, we conducted a linear regression on the participants’ payoffs based on their self-reported numbers. According to Table 1, the participants had an incentive to be dishonest and reported higher numbers (except six) to obtain a higher payoff. Therefore, participants who reported higher payoffs could be regarded as having a higher probability of lying.

We set a dummy variable Foreign Language that is equal to one if the participant was assigned to the FL condition and zero if he/she was assigned to the NL condition. Table 3 Column (1) reported the significant negative effect of FL on the payoff.

**Table 3:** Regression analysis (dependent variable: payoff).

Independent variable	(1)	(2)	(3)
Foreign language	−41.69^***^	−39.53^***^	−51.55
	(−4.44)	(−3.87)	(−0.85)
Gender		7.74	8.061
		(0.71)	(0.73)
Age		−3.51	−3.81
		(−0.93)	(−1.00)
English-score		−0.30	
		(−0.54)	
Foreign Language^*^English-score			0.15
			(0.20)
Constant	109.59^***^	199.78^**^	181.81^**^
	(15.92)	(2.40)	(2.40)
Obs	106	94	94
*R^2^*	0.1595	0.1702	0.1678

** *significant at 5% and*

*** *significant at 1%; t value in brackets.*

According to the existing literature, the lying behaviour of individuals is affected by some factors. There are obvious differences in lying behaviour between men and women, and men are more likely than women to lie (Fumagalli et al., 2010; MacDorman et al., 2010; Houser et al., 2012). Age also affects individuals’ lying tendencies. Friesen and Gangadharan (2012) find that the magnitude of dishonesty was significantly greater for older subjects. The variable English-Score is the score of participants in their English examination in the previous semester and adopted as a proxy for English language ability.^3^ In Column (2), gender, age and English-Score are regarded as control variables in the regression.

Table 3 Column (1) reveals that the effect of FL on payoff is significantly negative. Table 3 Column (2) reports the regression results based on the ordinary least squares (OLS) method. Although the coefficients of these control variables are not significant, the negative effect of FL on the payoff is still significant. According to the results, the condition dummy Foreign Language has a significant negative impact on payoff; after controlling for other factors, the payoff was 39.53 yuan less to participants in the FL condition than to those in the NL condition after controlling gender, age and their English language ability. The result proves that lying behaviour occurred significantly less often in the FL condition than in the NL group. The overall model is significant, which is consistent with the chi-square test results and further verifies that using a FL has a significant impact on decreasing lying behaviour.

These results were consistent with the cognitive load hypothesis, that is using a FL will increase cognitive load and make lying more difficult. Therefore, we can predict a smaller FL effect in high-ability participants than low-ability participants. In Table 3 Column (3), the interaction FL*English-score was introduced. The results showed that the coefficient of FL*English-score was positive which was contradictory to the coefficient of FL. Although both coefficients are not significant, the signs are consistent with our prediction.

In conclusion, the experimental results in Study 1 reveal that when age, gender and English ability were controlled for, more participants tended to misreport earning more money in their NL than in a FL, demonstrating that dishonesty is not language independent.

## Study 2: A Cheap-Talk Sender-Receiver Game

Study 1 revealed the difference in lying behaviour between the NL and FL conditions. However, with the design of Study 1, we can obtain the distribution of reported numbers revealed at the group level, but we cannot determine whether a subject lied or not. Besides, guilt is one of the emotions related to dishonesty and guilt a version will decrease lying behaviours. However, in the die-roll game, this kind of emotion might not be aroused, as participants may feel that they play the game alone and their choices have no effect on others. To better understand why speaking a FL reduces lying, we designed a cheap-talk sender-receiver game in which dishonesty could be observed at the individual level and there is an interaction between the participants and their counterparts, namely, the interaction between the senders and the receivers.

### Participants

Eighty-seven students (45 female) from different majors at the Zhejiang University were recruited *via* advertisements posted on the Internet. The average age of the participants was 23.13years. Their NL was Chinese, and they spoke English as a FL. The participants received RMB 5 as compensation for participating. Apart from the show-up fee, they would be paid according to their decisions in the experimental task, which was conducted online. Participants were randomly assigned to the NL condition (*n*=45) or the FL condition (*n*=42).

Before the beginning of the experiment, participants who were randomly assigned to the FL condition were required to finish a lexical test which was proposed by Lemhöfer and Broersma (2012). This test was used to obtain a general indication of their proficiency in English in terms of vocabulary knowledge.^4^ If they could not pass the test, they were not allowed to continue the following experimental task, as they would have difficulty in understanding the cheap-talk sender-receiver game in English. All the 42 participants reported in the FL condition passed the English language test.

After the experiment, all the participants completed a questionnaire related to their demographic information and English language ability. In the questionnaire, the participants rated their English ability on a 4-point scale, with 4 indicating full fluency (*M*=3.54, *SD*=0.64). The two groups showed no significant differences in English proficiency (Mann-Whitney test, *p*=0.6535).

### Materials and Procedure

#### Task

We designed a laboratory experiment, a cheap-talk sender-receiver game inspired by Gneezy (2005) to investigate the dishonesty of the participants when their lies could negatively impact on another person’s payoff. We adopted a between-subjects design to test whether speaking a FL changed the participants’ honesty rate by comparing their lying behaviour in the two conditions.

In the cheap-talk sender-receiver game, one sender was paired with one receiver. For each pair, there were two possible monetary distributions. Only the senders had private information about the real monetary consequences of the distributions, and they then decided to send an honest/dishonest message about the allocation to the receivers. In our study, all participants played the role of sender, and an experiment assistant played the role of receiver.

More specifically, in Study 2, the participants who acted as senders were informed about the monetary payoff allocation of two options, A and B, over 12 trials. Crucially, all senders knew that the receivers would never be informed of the real payoff allocation as follows.

The allocation of earnings (example):
Option A: You earn ¥10(X); he/she earns ¥7(Y)
Option B: You earn ¥7(M); he/she earns ¥10(N).
Notes: X>Y, M<N and X>M
The sender was required to decide which message was sent to the receiver.
Message 1: ‘Option A will earn you more money than Option B’.
Message 2: ‘Option B will earn you more money than Option A’.

In our design, the values of X, Y, M and N varied in the 12 trials; Option A always earned the sender more money than Option B, and Option B always earned the receiver more money than Option A. Therefore, Message 1 is false, and Message 2 is true. However, it was in the sender’s interest to send Message 1, which was defined as lying behaviour. As the receivers had no information about the two options, he/she just made the decision according to the received message. In this way, the sender’s payoff was determined by the sender’s message. For example, if the receiver received Message 1(2) and chose Option A(B), the sender obtained ¥X (¥M). At the end of the game, we randomly chose one of the 12 trials to determine the real payoff for the participants.

#### Experimental Procedure

**Figure 2:**
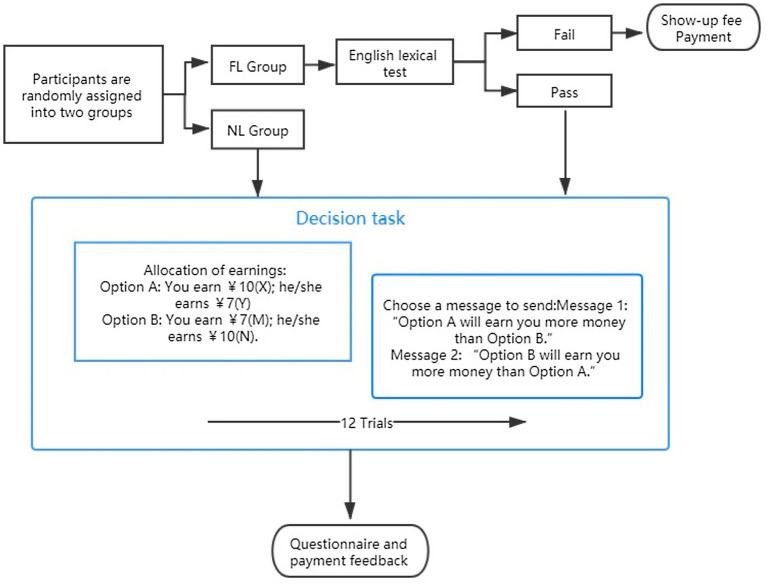
Procedure of the experiment.

Among 87 participants, 42 were randomly assigned to the FL condition, and 45 were assigned to the NL condition. The experiment was conducted online using o-Tree, which is an open-source platform for laboratory, online and field experiments (Chen et al., 2016). The procedure is shown in Figure 2. Sending Message 1 was defined as lying behaviour; sending Message 2 was defined as honest behaviour. In the questionnaire conducted after the experiment, we obtained demographic background data that included English self-assessment level and other variables for all participants.

### Results

We set the variable Dishonesty equal to 1 if a participant lied by sending Message 1 to his/her counterpart in one trial; otherwise, it equals 0. RT (Reaction Time) is defined as the time duration of one sender getting the payoff information of the projects and sending the message in one trial.

Table 4 shows the mean value of the RT and Dishonesty for the NL and FL conditions in the cheap-talk game. According to the results, reaction time was significantly longer in the FL condition than in the NL condition. Moreover, participants were more likely to choose to lie when using a FL than when using their NL.

**Table 4:** Mean reaction time and dishonesty of participants.

Variables	Condition	*M*	Mann-Whitney test (*p* value)
RT (Reaction time per trial)	NL	10.33s	0.061^*^
	FL	12.16s	
	total	11.21s	–
Dishonesty	NL	0.56	0.030^**^
	FL	0.50	
	total	0.53	–

* *significant at 10% and*

** *significant at 5%.*

**Figure 3:**
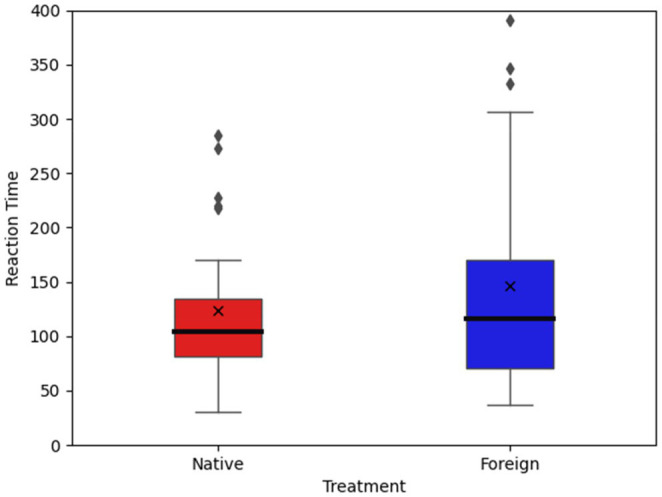
Total reaction time of participants.

**Figure 4:**
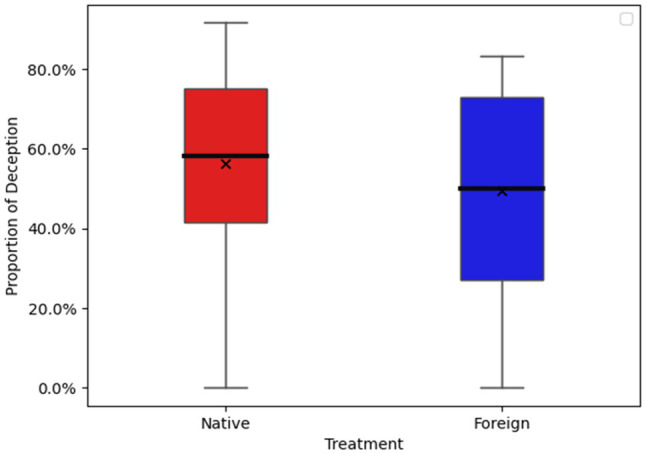
Proportion of participant lying behaviour.

The average RT and the average proportion of lying were statistically analysed by one-way ANOVA. The main effect of language was significant, *F*=27.63, *p*<0.001. The proportion of lying behaviour in the NL condition (53.07%) was significantly higher than that in the FL condition (49%). The main effect of language was significant, *F*=4.70, *p*=0.03. Figures 3, 4 show the total RT of 12 trials and proportion of lying in 12 trials for both groups.

To further explore the lying behaviour under two different language conditions, we separated the trials when participants choose to lie from the trials with honest decisions. On average in one trial, participants took 11.77s to lie when using a FL and 10.63s to lie when using their NL. The reaction time for lying was significantly longer under the FL condition than under the NL condition (Mann-Whitney test, *p*=0.07). However, under those two conditions, the reaction times for honest decisions did not differ (Mann-Whitney test, *p*=0.44). The results show that people need more time to lie in a FL because the cognitive load is higher under that condition.

Based on the cognitive load theory, we could predict that participants with lower FL ability will cheat less in the FL, as the cognitive load is stronger for them when using English. According to the number of correct answers in the English test before the task, we classified one participant as low-ability type if his/her number of correct answers was lower than 40 out of 60. Otherwise he/she was high-ability type. We calculated the frequency of lying for each participant in the whole 12 trials in the FL group. We found that the average frequency of lying was 50.91% for high-ability participants and 42.86% for low-ability participants (t test, *p*=0.011). It revealed that using foreign language increases the difficulty of lying.

To further verify the impact of using a FL on individual lying behaviour, we conducted a logistic regression on the variable Dishonesty, which was a binary outcome. Some factors that might be related to dishonesty are controlled in the regression. Besides the control variables that were similar to the control variables in Study 1 (Table 3), we also used trial as an independent variable. In total, we considered 1,044 observations with 87 subjects and 12 trials.

Table 5 Column (1) and (2) report the regression results based on the logistic regression method. The results show that the coefficient of FL is significantly negative. Although the decision task in Study 2 is different from that in Study 1, the results of these two experiments are consistent with each other. Both results provide evidence that using a FL reduces lying behaviour.

**Table 5:** Logistic regression analysis (dependent variable: Dishonesty).

Independent variable	(1)	(2)
FL	−0.269^**^ (−2.16)	−0.256^**^ (−2.01)
Gender		−0.170 (−1.34)
Age		−0.030 (−1.04)
Trial		−0.041^**^ (−2.29)
Constant		1.285^*^ (1.88)
Obs	1,044	1,044
Pseudo R^2^	0.0033	0.0091

** significant at 5%;

* significant at10%; and t value in brackets.

Based on the emotional distance hypothesis, message senders using a FL will feel less guilt towards message receivers. Thus, the probability of lying would be higher in the FL than in the NL. In Study 2, our results show the opposite. We found that fewer participants in the FL group chose to send false messages to their receivers to obtain a higher payoff than did those in the NL group. The results are consistent with Study 1, and both support cognitive load theory, which predicts less dishonest behaviour when using a FL.

## Conclusion

With the integration of the global economy, an increasing number of people need to work or live in an environment where a FL is spoken. It is of great significance to understand people’s decision-making behaviour in FL conditions. This paper explores the FL effect of lying behaviour, which could be linked to some activities in international trade or international finance. For example, in trading, sellers aim to over-report the quality of their products to earn more profit.

To answer the question of whether people behave differently under different language conditions, we investigated the behavioural characteristics and brain mechanisms of sophisticated lying among college students. We designed a simple die-roll task in Study 1 and a cheap-talk sender-receiver game in Study 2. Although the tasks differed in the two experiments, both sets of results revealed that fewer participants chose to lie when they used a FL than when they used their NL.

According to the emotional distance hypothesis, using a FL reduces emotionality associated with lying, e.g. guilt. Hence, people lie more when using a FL. However, our results provide some evidence to support cognitive load theory, which proposes that the cognitive load associated with thinking in a FL is a burden that makes lying more difficult. We observed less lying behaviour when the experiment was conducted in a FL. In Study 2, the results reveal that participants needed more time to decide to lie under the FL condition than under the NL condition. However, there was no significant difference in the number of honest decisions under these two conditions. These results might imply that participants had more difficulty lying in a FL than in their NL. This result is consistent with the findings of Bereby-Meyer et al. (2020). The discovery that people are more honest in a FL is predicted by the cognitive load hypothesis in that lying typically requires greater mental effort than telling the truth. Using a FL can increase cognitive load, thereby increasing the difficulty of lying.

Some limitations of our study should be noted. First, we adopted a between-subjects experimental design, and individual differences cannot be completely excluded. Second, due to the limitation of the participant group and experimental cost, the conclusion of this paper is applicable only to cases in which English is the FL.

## Data Availability Statement

The raw data supporting the conclusions of this article will be made available by the authors, without undue reservation.

## Ethics Statement

The studies involving human participants were reviewed and approved by the Ethics committee of Key Laboratory of Applied Brain and Cognitive Sciences, Shanghai International Studies University. The patients/participants provided their written informed consent to participate in this study.

## Author Contributions

XY, LL, and RL designed the experiment, wrote the manuscript, revised the manuscript, and finally approved the version to be published. LL performed the experiment and drew the figures. LL and RL analysed the data. All authors contributed to the article and approved the submitted version.

## Funding

This work was supported by the National Natural Science Foundation of China (Project71873089) and National Social Science Fund of China (Project 15CGJ023).

## Conflict of Interest

The authors declare that the research was conducted in the absence of any commercial or financial relationships that could be construed as a potential conflict of interest.

## Publisher’s Note

All claims expressed in this article are solely those of the authors and do not necessarily represent those of their affiliated organizations, or those of the publisher, the editors and the reviewers. Any product that may be evaluated in this article, or claim that may be made by its manufacturer, is not guaranteed or endorsed by the publisher.

## Footnotes

^1^The largest number of participants, 50.9%, chose ‘understand relatively well’; 39.8% chose ‘absolutely understand’; and none of them chose ‘absolutely do not understand’.

^2^We recruit subjects from the Shanghai International Studies University which is a university with about 70% female students and 30% male students. The gender distribution of our participants sample is close to the whole university.

^3^The scores are reported by the participants in the questionnaire. As some participants cannot remember their scores, there are some missing samples for this variable. The average score was 81.10 out of 100 for participants in the NL and 80.82 for participants in the FL.

^4^This test consists of 60 trials, in each of which participants see a string of letters on the computer screen. Their task is to decide whether this is an existing English word or not. They have 10 s to complete each trial. Once they can answer more than 60% of the total trials correctly, they pass the test and start the following task.
